# Post-tilleyite, a dense calcium silicate-carbonate phase

**DOI:** 10.1038/s41598-019-44326-9

**Published:** 2019-05-27

**Authors:** David Santamaria-Perez, Javier Ruiz-Fuertes, Miriam Peña-Alvarez, Raquel Chulia-Jordan, Tomas Marqueño, Dominik Zimmer, Vanessa Gutiérrez-Cano, Simon MacLeod, Eugene Gregoryanz, Catalin Popescu, Plácida Rodríguez-Hernández, Alfonso Muñoz

**Affiliations:** 1 MALTA-Departamento de Física Aplicada-ICMUV, Universidad de Valencia, 46100 Valencia, Spain; 2 DCITIMAC, Universidad de Cantabria, MALTA Consolider Team, 39005 Santander, Spain; 3 Centre for Science at Extreme Conditions and School of Physics and Astronomy, University of Edinburgh, EH9 3JZ Edinburgh, UK; 4 Institute of Geosciences, Goethe-University Frankfurt, 60438 Frankfurt am Main, Germany; 5 Atomic Weapons Establishment, Aldermaston, RG7 4PR Reading, UK; 6 Center for High Pressure Science Technology Advanced Research, 201203 Shanghai, China; 7 CELLS-ALBA Synchrotron, Cerdanyola del Vallès, 08290 Barcelona, Spain; 8 Departamento de Física, Instituto de Materiales y Nanotecnología, Universidad de La Laguna, MALTA Consolider Team, 38206 La Laguna, Tenerife, Spain

**Keywords:** Mineralogy, Condensed-matter physics

## Abstract

Calcium carbonate is a relevant constituent of the Earth’s crust that is transferred into the deep Earth through the subduction process. Its chemical interaction with calcium-rich silicates at high temperatures give rise to the formation of mixed silicate-carbonate minerals, but the structural behavior of these phases under compression is not known. Here we report the existence of a dense polymorph of Ca_5_(Si_2_O_7_)(CO_3_)_2_ tilleyite above 8 GPa. We have structurally characterized the two phases at high pressures and temperatures, determined their equations of state and analyzed the evolution of the polyhedral units under compression. This has been possible thanks to the agreement between our powder and single-crystal XRD experiments, Raman spectroscopy measurements and *ab-initio* simulations. The presence of multiple cation sites, with variable volume and coordination number (6–9) and different polyhedral compressibilities, together with the observation of significant amounts of alumina in compositions of some natural tilleyite assemblages, suggests that post-tilleyite structure has the potential to accommodate cations with different sizes and valencies.

## Introduction

With the possibility of efficient deep carbon ingassing during the last billion years of Earth’s history^1^, the study of the nature of stable forms of carbon in Earth’s mantle and their variation as a function of depth is an important area of investigation. At convergent boundaries, carbon enters into Earth’s mantle via subduction in the form of carbonates^2^. Depending on the chemical environment and the thermodynamical conditions, some carbonates may survive in subducting slabs, diving deeper into the mantle^3,4^. Melting phase relations of recycled oceanic crust recently reported suggest that slabs should undergo melting and loss of carbonate components at 300–700 km depths^5^. However, the form carbon takes depends on the redox conditions in inner Earth, which is defined in terms of oxygen fugacity of the environment. The carbon oxidation state is still poorly constrained and is debated at present, but recent studies considered that carbonates may get reduced to diamond below ~250 km depth^6,7^ due to a low oxygen fugacity^8^. Therefore, the structure and properties of compositionally relevant oxidized carbon-bearing phases at shallower depths (upper mantle) are of great interest for the geophysical community. In this sense, silicate-carbonates are potential host structures for carbon in Earth’s interior, particularly in the proximity of carbonate-rich subduction slabs.

Only a few minerals that contain both silicate and carbonate groups exist, three of them belonging to the CaO-CO_2_-SiO_2_ system: Ca_7_(SiO_4_)_3_(CO_3_) galuskinite^9^, Ca_5_(SiO_4_)_2_(CO_3_) spurrite^10^, and Ca_5_(Si_2_O_7_)(CO_3_)_2_ tilleyite^11^. They form in the metamorphic zone between limestones (sharp contact with marble, mainly composed of CaCO_3_ calcite) and igneous rocks (CaSiO_3_ wollastonite and other silicate minerals), at low pressures and high temperatures^12,13^. Significant aqueous fluid fluxes in marbles and calc-silicate rocks may result in the production of these types of minerals at lower temperatures, depending also on fluid composition^14^. Thermodynamical data and phase relations of minerals in this ternary system remain scarce^14,15^. Liu and Lin studied the phase stability of two of these calcium silicate carbonates, spurrite and tilleyite, using laser-heated diamond-anvil cells and found that these minerals decompose into their component silicates and carbonates above 4 GPa and ∼1000 °C^16^. On the other hand, experimental studies at low pressures suggest that spurrite and tilleyite have a field of stability between 600° and 1000 °C^12,17^. It is then fundamental to invest in a more detailed knowledge of these compounds at pressures and temperatures of deep subduction regions, since these silicate-carbonate minerals might be constituents of “cold” slabs. In this regard, it is important to attain an accurate description of the crystal structures and physical properties of these materials at high pressures and temperatures. The compressibility and phase stability of spurrite up to 27 GPa and 700 K have been recently reported^18^. No phase transformation was found, which suggests that the presence of carbonate [CO_3_] units gives stability to the tetrahedral [SiO_4_] orthosilicate group, holding up the formation of phases based on octahedral [SiO_6_] units. What occurs to tilleyite with a similar configuration remains unknown calling for further investigations.

Tilleyite is a naturally-occurring calcium silicate-carbonate mineral that conforms well with the chemical formula Ca_5_(Si_2_O_7_)(CO_3_)_2_, but some minor substitution of other elements such as Al, Mg or F could occur^12,19^. It is therefore the most carbonated compound of the calcium silicate-carbonate family. The existence of this mineral was firstly reported by Larsen and Durham in 1933 from contact altered limestones at Crestmore quarries, California, USA^11^. The monoclinic crystal structure of tilleyite was firstly determined by Smith^20^ and later refined by Louisnathan and Smith^21^, and Grice^22^. The structure contains the following atom-centered oxygen polyhedra as building blocks: [CO_3_] triangles, [SiO_4_] tetrahedra forming disilicate [Si_2_O_7_] units, and five irregular polyhedra with different coordination spheres for Ca atoms (20% are octahedrally coordinated, 60% are forming 7-fold-coordinated capped octahedra and 20% are forming distorted cubes). It is envisaged that the flexibility of the bridging Si–O–Si dihedral angle of the disilicate group upon compression could lead to an increase of the coordination number of the Ca atoms and the transition to a denser phase.

Here we present, in contrast to spurrite counterpart, the formation of a novel silicate-carbonate phase after compressing tilleyite above 8 GPa. The high-pressure polymorph is characterized *in situ* by X-ray diffraction (XRD) and Raman spectroscopy. DFT calculations confirm that the new phase is thermodynamically more stable at these conditions. We also found evidence for a second dense silicate-carbonate polymorph above 21 GPa. The initial tilleyite structure is recovered after pressure is quenched.

## Results

### Phase stability and determination of a high-pressure phase

**Figure 1:**
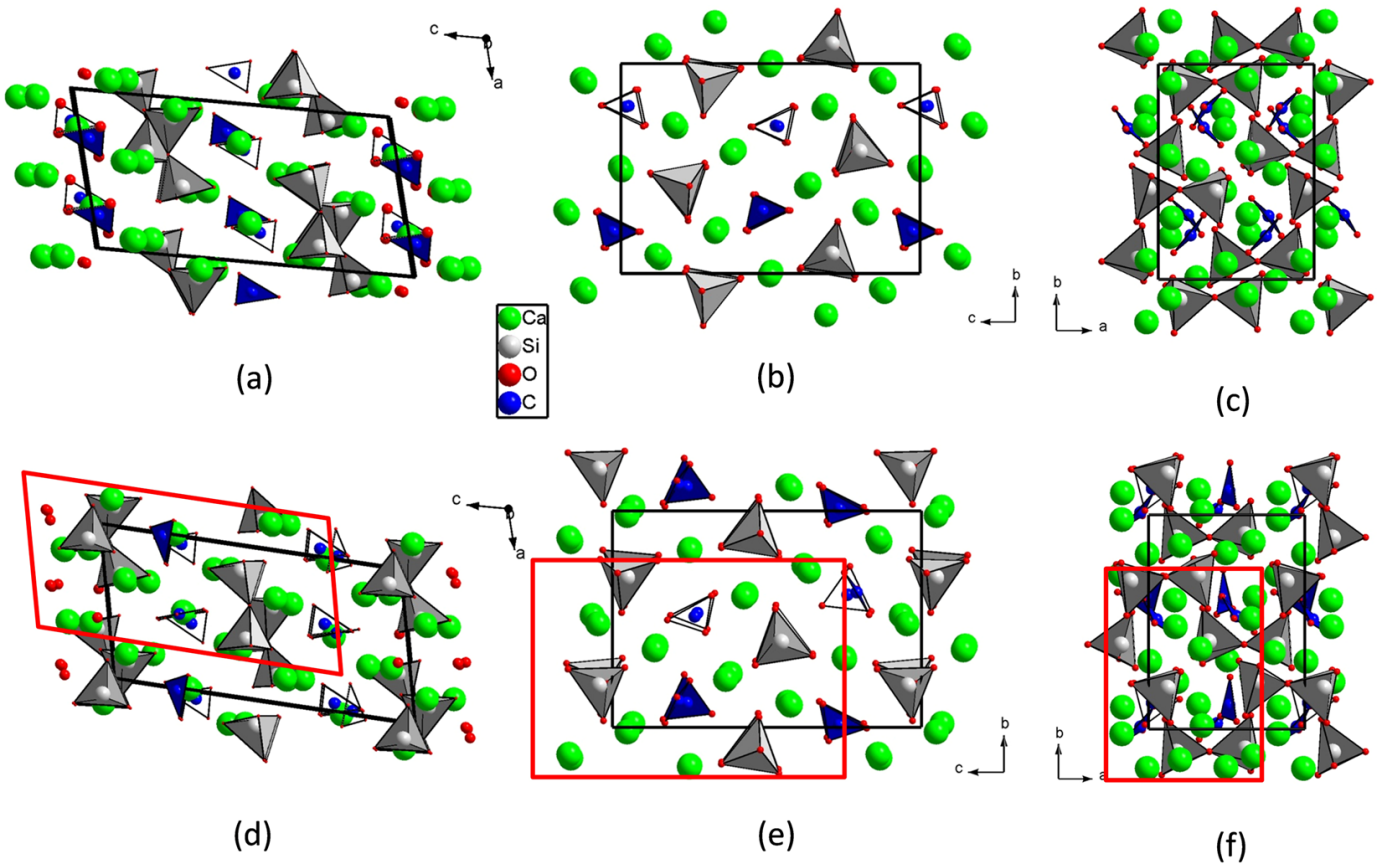
Structures of Ca_5_(Si_2_O_7_)(CO_3_)_2_ tilleyite (top) and high-pressure post-tilleyite (bottom) projected along the three unitcell axes. Blue, light gray, light green and red spheres represent C, Si, Ca and O atoms, respectively. Cell edges are depicted as solid black lines. Red solid lines on post-tilleyite projections demarcate the location of tilleyite unitcell contents, for the sake of comparison between structures.

Powder and single-crystal XRD measurements on tilleyite were performed under compression. The initial sample can be described with a monoclinic *P*2_1_/*n* space group and lattice parameters: *a* = 7.582(4) Å, *b* = 10.265(4) Å, *c* = 15.030(6) Å and *β* = 103.99(2)° (V = 1135.0(9) Å^3^, Z = 4), which are in excellent agreement with previously reported cell dimensions of other Crestmore specimens (once expressed in the same crystallographic setting). The structure is depicted in Fig. 1a–c and the atomic coordinates of the 22 independent atoms within the unit cell at ambient conditions are collected in Table 1 of the Supplementary Information, SI (to be compared with literature data of Table 2). The topological features of the structure are the same as previously determined, but a brief summary is needed to analyze subsequent structural transformations. In tilleyite, two [SiO_4_] tetrahedra share a common O atom to form a disilicate [Si_2_O_7_] group along the *a* axis and the [CO_3_] carbonate units are roughly oriented along the [001] direction. Ca polyhedra and disilicate units form corrugated walls parallel to (001) cross-linked by the [CO_3_] triangles. Voronoi-Dirichlet polyhedra of Ca atoms provide reliable information on the number of atoms that must be considered as first neighbors for bonding purposes^23^. There are five Ca sites with three types of coordination: 1 × [CaO_6_], 3 × [CaO_7_] and 1 × [CaO_8_]. Except the octahedron, the other Ca-centered polyhedra are very irregular and difficult to describe. Major distortions likely come from the shortening of edges shared between polyhedra, as expected from electrostatic interactions^21^. For instance, a result of the cooperative accommodation of the different building units is that the [Si_2_O_7_] groups flex to yield a dihedral angle of 156.86(14)°.

**Table 1:** Experimental details of the Ca_5_(Si_2_O_7_)(CO_3_)_2_ post-tilleyite single-crystal structure refinements at 10.8 GPa.

**Crystal data**	
Chemical formula	Ca_5_(Si_2_O_7_)(CO_3_)_2_
Crystal system	Monoclinic
Space group	*P*2_1_
Unit cell parameters	*a* = 7.3402(11) Å
	*b* = 9.7652(7) Å
	*c* = 14.354(9) Å
	*β* = 103.88(4)°
Cell volume	998.8(9) Å^3^
Z	4
Density	3.25 g/cm^3^
**Crystal structure refinement**	
Total number of reflections	6377
Unique reflections	5205
N°. refl. I > 3σ(I)	4355
R_int_	0.0578
R1	0.0553
R1_all_	0.0672
wR2	0.1355
N°. parameters	178
GooF	0.981

**Table 2:** Atomic coordinates and isotropic displacements (U_iso_) of Ca_5_(Si_2_O_7_)(CO_3_)_2_ post-tilleyite phase at 10.8 GPa.

Atom	x	y	z	U_iso_
Ca1	0.3197 (2)	0.11533 (14)	0.4325 (2)	0.0103 (2)
Ca2	0.1669 (2)	0.60264 (14)	0.5757 (2)	0.0104 (2)
Ca3	0.7990 (2)	0.35788 (14)	0.8650 (3)	0.0123 (3)
Ca4	0.32962 (19)	0.37492 (13)	0.8588 (2)	0.0105 (2)
Ca5	0.18118 (19)	0.22126 (13)	0.6343 (2)	0.0098 (2)
Ca6	0.9917 (2)	0.81712 (15)	0.7512 (3)	0.0120 (2)
Ca7	0.3427 (2)	0.01681 (14)	0.9344 (2)	0.0116 (2)
Ca8	0.33798 (19)	0.74761 (14)	0.3808 (2)	0.0104 (2)
Ca9	0.17152 (19)	0.47862 (14)	0.0473 (2)	0.0098 (2)
Ca10	0.5049 (2)	0.32289 (15)	0.2506 (2)	0.0116 (2)
Si1	0.8222 (3)	0.39301 (19)	0.4558 (3)	0.0080 (3)
Si2	0.1194 (3)	0.19132 (19)	0.0452 (3)	0.0083 (3)
Si3	0.6873 (3)	0.2176 (2)	0.0487 (3)	0.0083 (3)
Si4	0.3823 (3)	0.40791 (19)	0.4538 (3)	0.0080 (3)
C1	0.4829 (9)	0.6123 (6)	0.2392 (11)	0.0091 (10)
C2	0.0517 (9)	0.5120 (6)	0.7340 (11)	0.0100 (10)
C3	0.4380 (9)	0.0276 (6)	0.2744 (11)	0.0099 (10)
C4	0.0225 (10)	0.1234 (6)	0.7899 (12)	0.0106 (10)
O1	0.0383 (9)	0.4955 (6)	0.8220 (11)	0.0169 (11)
O2	0.9651 (7)	0.6079 (5)	0.6802 (9)	0.0112 (8)
O3	0.5793 (8)	0.3194 (6)	0.9685 (9)	0.0141 (10)
O4	0.2899 (7)	0.3394 (5)	0.3533 (9)	0.0113 (9)
O5	0.1753 (9)	0.2761 (6)	0.9601 (11)	0.0207 (12)
O6	0.9762 (10)	0.0206 (7)	0.8306 (11)	0.0247 (14)
O7	0.6201 (7)	0.0628 (5)	0.0379 (9)	0.0111 (9)
O8	0.8894 (7)	0.2128 (6)	0.0165 (9)	0.0119 (9)
O9	0.8125 (8)	0.3297 (6)	0.3525 (10)	0.0141(10)
O10	0.6115 (7)	0.3952 (5)	0.4787 (9)	0.0116 (9)
O11	0.1398 (7)	0.0303 (5)	0.0292 (9)	0.0113 (9)
O12	0.9159 (7)	0.2865 (5)	0.5385 (9)	0.0115 (9)
O13	0.3464 (7)	0.5647 (5)	0.4714 (9)	0.0116 (9)
O14	0.5296 (7)	0.1194 (5)	0.3309 (9)	0.0114 (9)
O15	0.8930 (7)	0.5467 (5)	0.4733 (9)	0.0117 (9)
O16	0.4528 (8)	0.0172 (6)	0.1873 (9)	0.0145 (10)
O17	0.3460 (7)	0.3062 (5)	0.5369 (9)	0.0103 (8)
O18	0.4366 (8)	0.5356 (6)	0.1639 (9)	0.0151 (10)
O19	0.5385 (8)	0.5618 (6)	0.3222 (10)	0.0145 (10)
O20	0.4781 (8)	0.7431 (6)	0.2324 (10)	0.0158 (10)
O21	0.3238 (7)	0.9526 (5)	0.3059 (9)	0.0108 (8)
O22	0.7111 (8)	0.2953 (5)	0.1487 (10)	0.0139 (10)
O23	0.1668 (8)	0.4378 (5)	0.7001 (9)	0.0130 (9)
O24	0.2063 (8)	0.2685 (6)	0.1446 (10)	0.0140 (10)
O25	0.9075 (9)	0.2151 (7)	0.7500 (11)	0.0253 (14)
O26	0.1988 (9)	0.1394 (6)	0.7916 (12)	0.0217(13)

**Figure 2:**
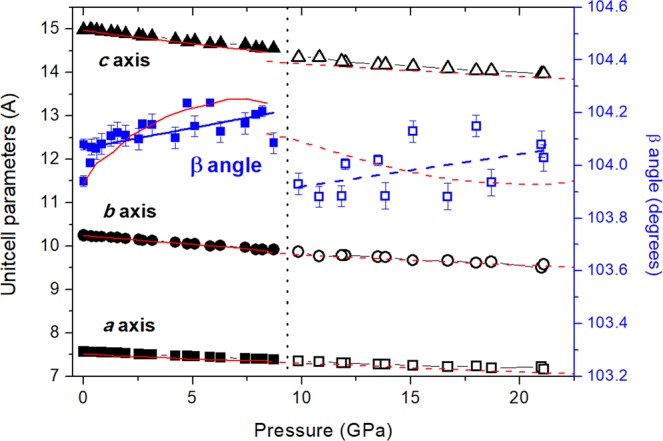
Pressure dependence of the lattice parameters of tilleyite and post-tilleyite Ca_5_(Si_2_O_7_)(CO_3_)_2_ phases. Black squares, circles and triangles denote the *a*, *b* and *c* axes of both structure-types. Blue squares show the evolution of the monoclinic β angle with pressure. Solid and empty symbols correspond to the initial *P*2_1_/*n* tilleyite and the high-pressure *P*2_1_ post-tilleyite phases. Lattice axes uncertainties are smaller than the size of the symbols. Error bars corresponding to the monoclinic β angle are indicated. Experimental fits are depicted as solid and dashed black and blue lines. Results from DFT calculations are represented with red lines.

**Figure 3:**
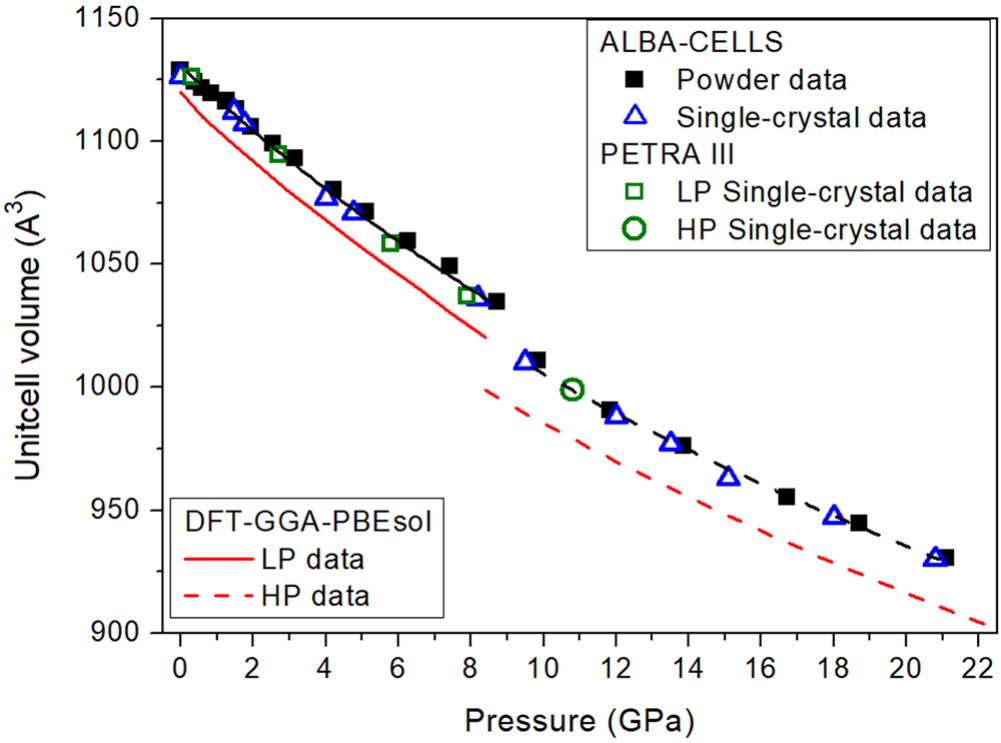
Pressure dependence of the unit-cell volume of Ca_5_(Si_2_O_7_)(CO_3_)_2_ tilleyite. Solid squares correspond to powder XRD data, and empty blue triangles and green circles correspond to single-crystal XRD data. Fits to all the experimental results for the low- and high-pressure phases are depicted as solid and dashed black lines, respectively. Results from DFT calculations are represented with red lines.

Powder diffraction data present intensities that do not correspond to perfect randomly oriented powder, so only peak positions and not relative intensities could be used to the structural analysis. In other words, from powder diffraction data, we could only accurately infer the lattice parameters of the mineral upon compression (see an example of LeBail refinement in Fig. 1 of SI). Indexations and profile fittings of the powder XRD patterns up to 22 GPa, the maximum pressure reached in this study, suggest that the structure can be described by a monoclinic space group and comparable unit cell dimensions in the whole pressure range. However, the evolution of the lattice parameters (Fig. 2), the monoclinic beta angle (Fig. 2), and unit-cell volume (Fig. 3) as a function of pressure evidence a clear discontinuity at 8–9 GPa. More specifically, according to our experiments (calculations), the *a* axis does not change noticeably, but *b* and *c* axes decrease at the transition about 0.2 (0.5) and 1.3 (1.5)%, respectively, and the *β* monoclinic angle suddenly decreases about 0.28 (0.12)°, giving as a result a unit cell contraction of about 1.5 (2.1)%. It is worth noting that the low symmetry and the large unit cell parameters preclude an unequivocal space group assignment, since numerous reflections overlap in powder XRD patterns. This problem is accentuated upon compression with the progressive broadening of the XRD reflections.

Single-crystal XRD measurements allow us to fully characterize the progressive transformations of the tilleyite structure with increasing pressure and the nature of the high-pressure silicate-carbonate phase. Our results show that in tilleyite there is a Ca–O distance that rapidly decreases upon compression, increasing the formal coordination number of one of the seven-fold coordinated Ca atoms to eight for pressures above 6 GPa (see crystallographic data of tilleyite at 7.9 GPa in Table 3 of SI). Above 9 GPa, the crystal structure can no longer be described by the tilleyite *P*2_1_/*n* space group, but by the lower symmetry *P*2_1_ space group. The structure was solved with a dataset at 10.8 GPa that has a reciprocal space completeness of 26.7% up to a resolution of 0.46 Å. The position of the Ca and Si atoms was determined by SHELXT^24^. Subsequent structural refinement and analysis of the difference-Fourier maps of the electron density function afforded localizing the rest of atoms of the unit cell. The final refinement of the structure was performed with SHELXL^25^ and included all the atomic coordinates and the isotropic displacement parameters. Data pertinent to the intensity data collection are given in Table 1 and the final positional parameters of post-tilleyite at 10.8 GPa are collected in Table 2.

Figure 1d–f show the projections of the high-pressure phase, hereafter named post-tilleyite, along the crystallographic axes. Although the transition comes with a volume collapse of ∼1.5%, the symmetry of the initial and final structures is related by a group-subgroup relationship and they can be easily compared (see Fig. 1a–f). The coordination spheres around the C and Si atoms by O atoms have not changed, remaining in trigonal planar and tetrahedral configuration. However, in contrast to tilleyite, the [CO_3_] groups in post-tilleyite are no longer coplanar with each other. Half of them maintain their initial orientation whereas ¼ are now roughly perpendicular to the *c* axis and ¼ are roughly perpendicular to the *a* axis. A similar rotation of carbonate groups was observed in the high-pressure polymorph of calcite, CaCO_3_-III^26^. This fact supports previous descriptions of tilleyite in terms of silicate and carbonate separated layer modules, where the CaCO_3_ component seems to follow a crystal behavior comparable to that of calcite itself in the same pressure range (CaCO_3_-III is reported to be stable between 2.5 and 15 GPa).

Post-tilleyite also contains two types of disilicate groups due to symmetry reduction, which causes that the Wyckoff positions 4e in the tilleyite *P*2_1_*/n* structure split into two symmetrically independent 2a positions. The dihedral angle of the [Si_2_O_7_] units change at the transition from 154.3° at 7.9 GPa to 149.4° and 156.7° at 10.8 GPa. These topological transformations entail considerable changes in the coordination number of Ca atoms. For instance, at 10.8 GPa, analyses of Voronoi-Dirichlet polyhedra reveal that Ca–O distances between 2.16 and 2.88 Å make up the Ca first-coordination spheres in post-tilleyite, defining variable Ca coordination polyhedra in different sites. The Ca coordination number varies from 6 to 9 (1 × [CaO_6_], 3 × [CaO_7_], 5 × [CaO_8_], 1 × [CaO_9_]). The existence of such a range of different polyhedral volumes within the same structure is very unusual and interesting from the geophysical point of view. Such a structure could likely host divalent cations with different radii without inducing significant elastic strains. This is particularly relevant within the complex scenario of Earth’s mantle, where natural compositions likely include Mg^2+^, Fe^2+^, and other cations coexisting with Ca^2+^. Thus, post-tilleyite offers a versatile structural possibility in comparison with aragonite, the most stable structure for Ca-rich carbonates in the upper and intermediate mantle, which has large polyhedral voids to host small cations (Ca atoms in nine-fold coordination)^27^.

**Figure 4:**
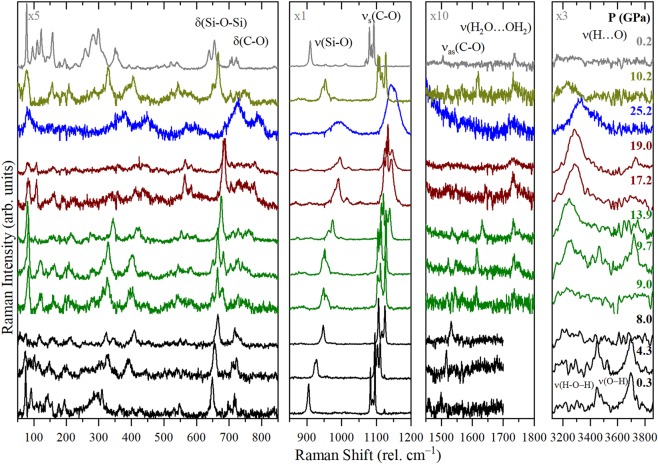
Selected room-temperature Raman spectra of powder Ca_5_(Si_2_O_7_)(CO_3_)_2_. Pressures are indicated at the right-hand side. Intensity scales vary from spectral regions and it has been adapted for clarity as indicated in the top left side of each panel. Black is used for tilleyite, green for post-tilleyite, and blue for higher pressure phase. δ symbols denote bending modes and ν stretching modes, respectively.

To further understand the structural evolution of tilleyite under compression, we performed *in situ* Raman spectroscopy measurements up to 25 GPa. Group theoretical analyses (point group C_2h_) predict 132 Raman active modes (Γ_R_ = 66A_g_ + 66B_g_) and a complicated Raman spectrum^28^. Raman scattering spectra at some representative pressures are shown in Fig. 4 (All spectra in Fig. 2 of SI). The large number of modes of low-pressure Ca_5_(Si_2_O_7_)(CO_3_)_2_ tilleyite precludes mode identification and only a rough assignment of fundamental vibrational modes was done. They can be distributed according to the following grouping: librational modes involving Ca^2+^ and [SiO_4_] and [CO_3_] units below 400 cm^−1^; bending modes of [SiO_4_] and [CO_3_] units between 400 and 780 cm^−1^; internal stretching of the [SiO_4_] and [CO_3_] groups in the 800–1500 cm^−1^ range, and OH stretching region above 3000 cm^−1^.

The most intense bands appear between 900 and 1100 cm^−1^. The band at 905 cm^−1^ corresponds to the Si-O stretching of the tetrahedral units^29^. The intense triplet between 1083 and 1093 cm^−1^ corresponds to the symmetric C-O stretching from the [CO_3_] units. The triplet structure is a consequence of the different environment surrounding the two distinct non-equivalent [CO_3_] units of tilleyite. In the lower frequency region, at 649 cm^−1^, the bending of the Si-O-Si inter-tetrahedral linkage appears^30–32^. The bands between 697 and 780 cm^−1^ are assigned to the [CO_3_] bending mode. In the low frequency region, the broad band at around 300 cm^−1^ is likely related to Ca-O connections, in analogy with the assignment done for wollastonite^32^. On the higher frequency region at around 1500 cm^−1^ the antisymmetric C-O stretching mode appears. Moreover, at around 3500 and 3700 cm^−1^, we observe two broad bands corresponding to O-H stretching mode of water and intersticial OH^28^. Due to the low intensity of these OH modes they were not always detected.

In Fig. 4 it can be seen the general upshift of the bands of tilleyite with increasing pressure (see also Fig. 2 of SI). No significant broadening is observed as expected by the crystallinity of the solid and the hydrostaticity provided by the pressure transmitting medium. In the low frequency region there are changes in the relative intensities of the modes that could be explained by the continuous change towards a higher coordination number of the Ca atoms induced by pressure, as revealed in XRD experiments.

Interestingly, between 8–9 GPa there is an abrupt splitting of the Si-O band and an even more remarkable one of the C-O bands, which perfectly agree with XRD results of the tilleyite to post-tilleyite transition. The orientational change of the [CO_3_] groups explains the splitting of the C-O stretching as its environment has changed. The splitting of the Si-O stretching into two contributions is perfectly explained by the existence of two types of unequivalent disilicate groups [Si_2_O_7_] in post-tilleyite while in tilleyite there was only one. In the bending mode regions between 600 and 800 cm^−1^, the Si-O bending splits into several contributions and some of them blueshift as a consequence of the observed decrease in the Si-O-Si bond angle^31^. On the other hand, the prints from the lattice modes drastically change as there appears an intense band at around 80 cm^−1^. The splitting of all the contributions at the phase transition is illustrated in Fig. 2 of the SI. Moreover, there is a change in the O-H stretching region at the transition as a new contribution appears at around 3000 cm^−1^. The presence of O-H stretching and bending bands provides evidence for the existence of [SiO_3_(OH)] groups in both, the low- and high-pressure phases. In addition to that aforementioned, the spectroscopic results presented in Fig. 4 show a significant band broadening between 19 and 25 GPa, which is a consequence of the abrupt increase of deviatoric stresses in the sample due to the fact that argon is highly non-hydrostatic above that pressure. Pressure-induced amorphization is ruled out because post-tilleyite and tilleyite spectra were observed on pressure release.

**Figure 5:**
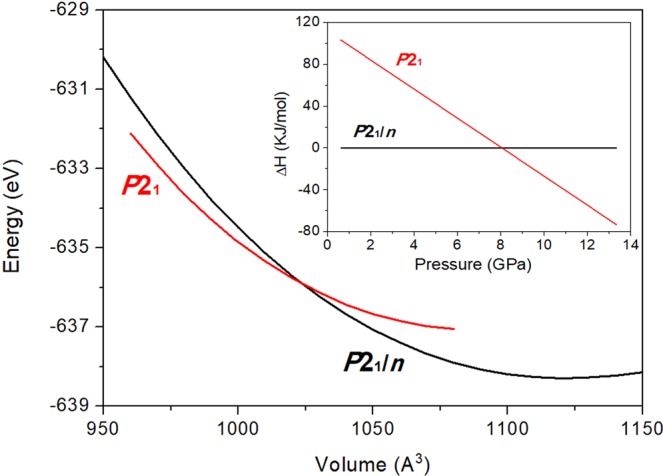
Internal energy as a function of volume per unitcell for the initial *P*2_1_*/n* tilleyite and the *P*2_1_ post-tilleyite phases. The enthalpy variation *versus* pressure curve for both polymorphs is depicted in the inset (taking the *P*2_1_*/n* tilleyite structure as reference).

Theoretical DFT calculations confirm that post-tilleyite is a thermodynamically more stable structure at high-pressures. Figure 5 shows the energy as a function of volume curves for the initial tilleyite and high-pressure post-tilleyite calculated structures. It can be clearly seen that both curves cross each other at high pressure. As shown in the enthalpy as a function of pressure graph of Fig. 5 inset, the denser phase becomes more stable at 8 GPa, in excellent agreement with the experimental data. Structural data from DFT calculations are also collected in Supplementary Information. Our simulations also provide frequencies and pressure coefficients of the IR- and Raman-active optical vibrations and, interestingly, show that some low-frequency modes corresponding to the tilleyite phase become softer with increasing pressure and eventually vanish at the phase transition pressure (Fig. 3 of SI). Furthermore, our theoretical data predict that post-tilleyite will be dynamically unstable above 21 GPa (Fig. 4 of SI).

Figures 2 and 3 illustrate the compression dependence of the lattice parameters and unit-cell volumes of both, tilleyite and post-tilleyite phases. The experimental and theoretical values are in good agreement and show a smooth and monotonous decrease under pressure. The strong anisotropy of tilleyite is described by the approximate linear axial compressibility values: β_a_ = 2.84(3)·10^−3^, β_b_ = 3.79(8)·10^−3^ and β_c_ = 3.48(9)·10^−3^ GPa^−1^ for the *a*, *b*, and *c* axes, respectively. These experimental axial compressibilities agree well with theoretical results: β_a_ = 2.8(1)·10^−3^, β_b_ = 3.92(5)·10^−3^ and β_c_ = 4.00(6)·10^−3^ GPa^−1^. Whereas the *b* and *c* lattice parameters show a quite similar relative contractions, the *a* axis is prominently harder (large incompressibility and rigidity of the [Si_2_O_7_] disilicate units), which means that the bulk compressibility will be dominated by that of the *b* and *c* axes. The least-squares fit of a second-order Birch−Murnaghan equation of state (BM-EoS)^33^ to our experimental (theoretical) pressure-volume data of the initial phase give a zero-pressure unitcell volume, V_0_ = 1131(1) Å^3^ (1119.5(6) Å^3^) and a bulk modulus, B_0_ = 80(2) GPa (75.3(4) GPa). These values reflect a slightly lower compressibility than those reported by Gao and coworkers: V_0_ = 1168.90(2) Å^3^, B_0_ = 69.7(3) GPa, and B’_0_ = 4.0(1) from DFT calculations using the PBE prescription^34^. Post-tilleyite is only slightly more incompressible with BM-EoS characteristic parameters: V_0_ = 1110(4) Å^3^ (1088.7(4) Å^3^) and B_0_ = 83(3) GPa (82.1 (3) GPa), according to experimental (theoretical) results. Linear axial compressibilities indicate that the high-pressure phase is more isotropic: β_a_ = 2.3(2)·10^−3^ GPa^−1^ (2.64(3)·10^−3^ GPa^−1^), β_b_ = 2.5(2)·10^−3^ GPa^−1^ (2.31(5)·10^−3^ GPa^−1^) and β_c_ = 2.2(1)·10^−3^ GPa^−1^ (2.00(4)·10^−3^ GPa^−1^) for the *a*, *b*, and *c* axes, respectively.

**Figure 6:**
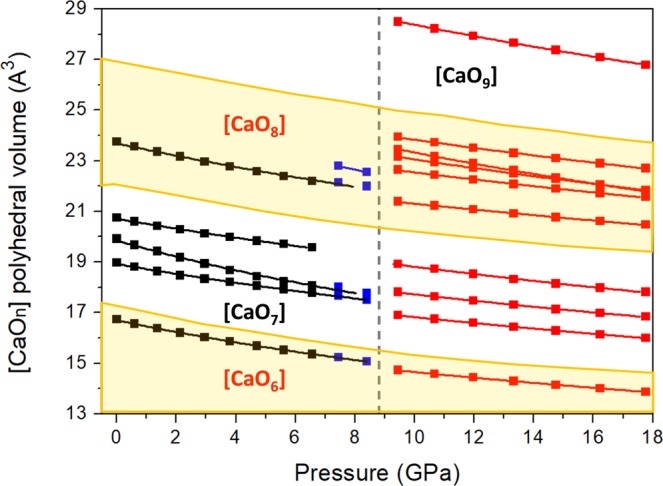
Representation of the evolution under compression of polyhedral unit volumes.

The evolution of the DFT-calculated polyhedral unit volumes with compression is shown in Fig. 6. The compression of the unit cell is dominated by the compression of the softer calcium-centered polyhedral units. In the case of the low-pressure tilleyite phase, two types of polyhedra have a lower bulk modulus than the unit cell, a seven-fold [CaO_7_] capped-octahedron and the [CaO_6_] octahedron, with B_0(P1)_ = 54.6(9) and B_0(P2)_ = 66.2(6) GPa, respectively (Table 6 of SI). After the transition, the remaining [CaO_6_] octahedron exhibits a similar bulk modulus B_0(P3)_ = 89.0(9) GPa to that of the unit cell, but two other polyhedral units are more compressible under compression: an 8-fold and the 9-fold coordinated Ca units with 62.7(3) and 83(1) GPa, respectively. Note that the diversity of environments of the Ca atoms is not only defined by the number of oxygen neighbours but also by the range of compressibilities of the different [CaO_n_] polyhedra, with bulk moduli as high as 139 (3) GPa.

Tilleyite was also studied at high-pressure high-temperature. The sample was progressively compressed and heated up to 7.2 GPa and 402 °C while characterized collecting *in situ* powder XRD data. The HP-HT patterns were indexed using the isolated single reflections and confirmed that the tilleyite structure remained stable in that P-T range (Table 7 of SI). Due to the fact that the pressure is not constant during heating, we used a perturbational method to estimate the thermal expansivity at low pressures, as described in ref.^18^. This approximation has been successfully used to calculate the thermal expansivity under compression in other compounds^35,36^. A linear fit to our data yields a volumetric thermal expansion value of 3.5(4)·10^−5^ K^−1^ (see Fig. 5 of SI). The thermal expansivity of tilleyite lies between those of CaCO_3_ aragonite (6.53·10^−5^ K^−1^) and CaCO_3_ calcite (2.01·10^−5^ K^−1^)^37^, and it is only slightly smaller than those of calcium silicate β-Ca_2_SiO_4_ larnite (4.24(4)·10^−5^ K^−1^)^38^ at room pressure and Ca_5_(SiO_4_)_2_(CO_3_) spurrite at ∼8 GPa (4.1(3)·10^−5^ K^−1^)^18^. CaSiO_3_ wollastonite, another end-member calcium silicate, exhibits a smaller thermal expansion (5.7–7·10^−6^ K^−1^)^39^. This fact suggests therefore that the thermal expansivity of Ca_5_(Si_2_O_7_)(CO_3_)_2_ tilleyite (2CaCO_3_ + Ca_2_SiO_4 + _CaSiO_3_ stoichiometric content) is a consequence of the structural homologies in the atomic arrangements with other members of the Ca-Si-C-O system.

## Discussion

The present study using a combination of powder and single-crystal XRD, Raman spectroscopy and DFT calculations clearly shows the existence of a novel silicate-carbonate polymorph under high P-T conditions. At 8 GPa, tilleyite, a naturally-occurring silicate-carbonate mineral transforms into a denser phase which presents a large variety of environments for Ca atoms, from six-fold to nine-fold coordination. The different classes of irregular cation-centered oxygen polyhedra exhibit a wide range of bulk moduli, which suggest that such a structure could accommodate other divalent cation species with different radii; *i*.*e*. Mg^2+^, Fe^2+^, Mn^2+^, etc…. On top of that, systematic studies on tilleyite reveal that traces of alumina and fluoride favor its formation, in agreement with compositions of natural mineral assemblages^12^. Synthetic tilleyite has been grown from starting material with up to 8.2 and 4.7% of Al_2_O_3_ and CaF_2_, respectively. This means that partial substitution of Si by Al atoms is possible and alkali metals of different sizes can also be hosted in both tilleyite and post-tilleyite structures. Our Raman data also shows the replacement of some oxygen atoms by OH groups. Note that potential chemical substitutions and the low symmetry of the post-tilleyite structure are envisaged to further stabilize this phase at high temperature via an increase of the entropic term.

The thermodynamic conditions for formation and preservation of Ca_5_(Si_2_O_7_)(CO_3_)_2_ tilleyite are under debate, but it seems to be stable within the 450° ^17^–970 °C^12^ temperature range at low pressures (<0.2 GPa). At 4 GPa, tilleyite is reported to break down at 1000 °C into a mixture of calcium carbonate and silicate phases: aragonite, wollastonite and larnite^16^. Accurate phase relation constraints at pressures higher than 0.2 GPa and temperatures lower than 1000 °C are required to better understand the complex CaO-SiO_2_-CO_2_ phase diagram and define the geological formation of this mineral. This study reports valuable crystallographic data for the high-pressure polymorphic transition of tilleyite which contribute to the understanding of silicate-carbonate crystal chemistry.

## Methods

### Initial samples

Naturally occurring tilleyite crystals from the Crestmore quarry in the Jurupa mountains (Riverside County, California, USA) were kindly provided by the Yale Peabody Museum (Specimen YPM MIN 041104). A few crystals were optically selected under the microscope and crushed with a mortar and pestle, giving a fine white powder without lustre. Qualitative chemical analyses were done on a Philips XL30 scanning electron microscope by the method of energy dispersive x-ray spectroscopy. Only traces (<1 wt%) of Al and K were found apart the Ca, Si, C and O atoms present in the ideal Ca_5_(Si_2_O_7_)(CO_3_)_2_ formulae. This is in good agreement with tilleyite chemistry reported in ref.^11^. for a tilleyite specimen collected in the same area. Angle-dispersive x-ray diffraction measurements at ambient conditions confirm the tilleyite structure.

### High-pressure methods

We performed high pressure (HP) experiments at both, room (RT) and high temperature (HT) conditions and the sample was characterized *in situ* by means of synchrotron angle-dispersive x-ray diffraction measurements. See details on pressure devices and sample configuration below.

### High-temperature methods

For the HP resistive-heating studies, the DAC was contained within a custom-built vacuum vessel designed for HP–HT experiments^40^. The DACs were heated using Watlow 240 V (rated at 4.65 W cm^−2^) coiled heaters wrapped around the outside of the DACs. The temperature was measured using a K-type thermocouple attached to one of the diamond anvils, close to the gasket. The accuracy of the thermocouple on the temperature range covered by the experiments is 0.4%^35,36^. NaCl powder was included in the sample chamber to act as pressure transmitting medium and pressure marker^41^.

### Powder XRD methods

HP-RT XRD experiments to 21 GPa were carried out in a gas-membrane-driven diamond-anvil cell (DAC) equipped with diamonds of 350 µm of culet diameter. The tilleyite sample was placed together with two ruby chips at the center of a 100 µm diameter 40 µm thick pressure chamber drilled in a preindented rhenium gasket. High-purity Ne gas was also loaded in the DAC at room temperature using the Sanchez Technologies gas loading apparatus. Ne is fluid up to 4.7 GPa and acted as a quasi-hydrostatic medium in the pressure range of the study^42,43^. Pressure was determined using the ruby fluorescence method^44^, which provided pressure accuracies of 0.1 GPa below 14 GPa and 0.2 GPa up to 23 GPa. The equation of state of Ne was used as a second pressure gauge^45^ above 5 GPa. *In situ* angle-dispersive powder X-ray diffraction measurements were carried out at MSPD beamline at ALBA-CELLS synchrotron using a x-ray wavelength of 0.534 Å (Rh K edge)^46^. This beamline provides a X-ray beam focused down to ~20 × 20 µm^2^, and the diffracted signal was collected with a Rayonix CCD detector. The detector parameters were calibrated with LaB_6_ powder standard, and integration to conventional 2*θ*-intensity data was carried out with the Dioptas software^47^. The indexing and refinement of the powder patterns were performed using the Chekcell^48^, Unitcell^49^ and Powdercell^50^ program packages.

### Single crystal XRD (SXRD) methods

Experiments up to 21.1 GPa were performed with a Boehler-Almax diamond anvil cell (DAC) (4θ~70°) in which a 10-µm-thick sample was introduced inside a 150 µm diameter hole of a stainless-steel gasket preindented to 40 µm. The pressure calibrant was a ruby chip and the pressure transmitting media were Ne and a 4:1 methanol:ethanol mixture. These experiments were done at the MSPD beamline at ALBA-CELLS synchrotron using a x-ray wavelength of 0.3185 Å (Ne as PTM) and at the P02.2 Extreme conditions beamline at PETRA III synchrotron, DESY, using a x-ray wavelength of 0.28973 Å (methanol:ethanol mixture as PTM). The maximum pressure in single-crystal runs was 21 GPa at ALBA-CELLS and 10.8 GPa at PETRA III. It has to be stressed out that few measurements resulted in succesful structural solution and satisfactory refinement relies (data measured at PETRA III). The diffraction images were collected by 0.5° ω scanning. The image format was converted for further processing with the CRYSALIS^*Pro*^ software^51^ for indexing reflections and intensity data reduction. The structures were refined with SHELXL^25^, operated using the WinGX interface^52^.

### Raman spectroscopy methods

Raman experiments were performed to 25.2 GPa using a symmetric screw-driven diamond-anvil cell equipped with ultra low-fluorescence diamonds of 200 μm culet diameter. A rhenium gasket was used to hold the sample, with a chamber of about 75 μm diameter and 30 μm thick. A Tilleyite powder sample was loaded in the chamber together with a ruby chip whose fluorescence is used as pressure calibrant. Argon gas was loaded into diamond anvil cells at a pressure of 0.2 GPa to be used as pressure transmitting medium. High-quality Raman spectra were acquired using a custom-built micro-focused Raman system, using a 514 nm laser as the excitation line. Raman scattering radiation was collected in back-scattering configuration. The device is equipped with a 20x Mitutoyo long working distance objective and focused onto the slit of a Princeton monochromator with a grating of 1800 gr/mm and a CCD detector.^53^ Spectra were measured with a spectral resolution of about 1–2 cm^−1^ and calibrated with a standard neon emission lamp. The typical sampling area was about 2 μm in diameter.

### Computational methods

The present study was performed through total energy ab initio simulations with the Vienna ab initio simulation package (VASP) plane-wave pseudopotential program^54–56^. We have employed the Born-Oppenheimer approximation and the electrons were quantum-mechanical treated by solving the Khon-Sham equations of density functional theory (DFT)^57^. The exchange-correlation energy was described with the generalized gradient approximation (GGA), according to the Perdew-Burke-Ernzerhof for solids (PBEsol)^58^ prescription. The projector augmented wave (PAW)^59,60^ was used to take into account the electron-ion interactions, and the plane waves basis set was extended up to a cutoff energy of 530 eV in order to obtain accurate results. The Brillouin zone integrations were carried out by means of a grid of Monkhorst-Pack^61^ (4 × 2 × 2) k-special points that provided well converged results due to the large size of the primitive cell, that contains 88 atoms, with a great number of degrees of freedom. For a set of different volumes, we achieved a total relaxation of all these free degrees to obtain the optimized structural parameters. The total energy was converged to 0.001 eV, the Hellman-Feynman forces on the atoms were lower than 0.004 eV/Å, and hydrostatic conditions were obtained, it means that the stress tensor was diagonal with an accuracy of 0.1 GPa. After these simulations for the set of volume-fixed cells, we got the volumes, total energies and pressures, as well as the cell parameters and the fractional atomic coordinates of the atoms. This allowed using an equation of state, EOS, to fit the data and to obtain the bulk modulus and its derivatives. This method was applied with very good results to the study of phase stability of many semiconductors compounds under high pressure^62^.

## Supplementary information

Supplementary information
