# Supplementary material for: Post-tilleyite, a dense calcium silicate-carbonate phase
Source: Sci Rep. 2019 May 27;9:7898. doi: 10.1038/s41598-019-44326-9 (PMC6536543; doi:10.1038/s41598-019-44326-9)

Post-tilleyite, a dense calcium silicate-carbonate phase

David Santamaria-Perez[1,*], Javier Ruiz-Fuertes[2], Miriam Peña-Alvarez[3], Raquel Chulia-Jordan[1], Tomas Marqueño[1], Dominik Zimmer[4], Vanessa Gutiérrez-Cano[2], Simon MacLeod[3,5], Eugene Gregoryanz[3,6], Catalin Popescu[7], Plácida Rodríguez-Hernández[8], Alfonso Muñoz[8]

[1] MALTA-Departamento de Física Aplicada-ICMUV, Universidad de Valencia, 46100, Valencia, Spain.
[2] DCITIMAC, Universidad de Cantabria, MALTA Consolider Team, 39005, Santander, Spain.
[3] Centre for Science at Extreme Conditions and School of Physics and Astronomy, University of Edinburgh, Edinburgh EH9 3JZ, UK
[4] Institute of Geosciences, Goethe-University Frankfurt, 60438 Frankfurt am Main, Germany
[5] Atomic Weapons Establishment, Aldermaston, Reading, RG7 4PR, UK
[6] Center for High Pressure Science Technology Advanced Research, 201203 Shanghai, China
[7] CELLS-ALBA Synchrotron, Cerdanyola del Vallès, 08290, Barcelona, Spain
[8] Departamento de Física, Instituto de Materiales y Nanotecnología, Universidad de La Laguna, MALTA Consolider Team, 38206 La Laguna, Tenerife, Spain.

* Correspondence to David.Santamaria@uv.es

SUPPLEMENTARY MATERIAL

Table 1a.- Experimental details of the $Ca_5(Si_2O_7)(CO_3)_2$ tilleyite single-crystal structure refinements at ambient conditions.

Crystal data	
Chemical formula	$Ca_5(Si_2O_7)(CO_3)_2$
Crystal system	Monoclinic
Space group	$P2_1/n$
Unit cell parameters	*a* = 7.582(4)Å
	b = 10.265(4)Å
	c = 15.030(6)Å
	β =103.99(2)º
Cell volume	1135.0(9)$Å^3$
Z	4
Density	2.859 g/cm^3
Crystal structure refinement	
Total number of reflections	65488
Unique reflections	12973
Nº. refl. I > 3σ(I)	7208
R_{int}	0.1573
R1	0.0696
$R1_{all}$	0.1408
wR2	0.1727
Nº. parameters	90
GooF	1.024

Table 1b.- Atomic coordinates of tilleyite at ambient conditions (Our data)

Atom	x	y	z
Ca1	0.92115(6)	0.08935(4)	0.87583(3)
Ca2	0.07275(6)	0.20638(3)	0.68114(3)
Ca3	0.24687(7)	0.00601(4)	0.49659(3)
Ca4	0.07896(7)	0.57889(4)	0.63623(3)
Ca5	0.91441(7)	0.71333(4)	0.82319(3)
Si1	0.56311(9)	0.92090(6)	0.70173(4)
Si2	0.13533(9)	0.91420(6)	0.70391(4)
C1	0.1913(3)	0.3088(2)	0.5301(2)
C2	0.2952(3)	0.6975(2)	0.47759(14)
O1	0.4015(3)	0.6241(2)	0.44422(14)
O2	0.2801(3)	0.6801(2)	0.55902(15)
O3	0.0985(3)	0.7644(2)	0.72697(12)
O4	0.6598(3)	0.0305(2)	0.77505(12)
O5	0.0898(3)	0.3766(2)	0.56967(14)
O6	0.0986(3)	0.0210(2)	0.77791(12)
O7	0.2162(3)	0.7950(2)	0.42843(12)
O8	0.6369(3)	0.7742(2)	0.72528(12)
O9	0.0498(3)	0.9719(2)	0.60272(12)
O10	0.2744(3)	0.2082(2)	0.57406(12)
O11	0.3600(3)	0.9176(2)	0.72565(13)
O12	0.2028(3)	0.3353(2)	0.44976(16)
O13	0.5433(3)	0.9762(2)	0.59945(13)

Table 2.- Atomic coordinates of tilleyite at ambient conditions from Grice (2005) [22] in the setting $P2_1/n$, for the sake of comparison.

Atom	x	y	z
Ca1	0.9211	0.0893	0.8758
Ca2	0.0726	0.2065	0.6812
Ca3	0.2468	0.0060	0.4966
Ca4	0.0789	0.5788	0.6362
Ca5	0.9144	0.7135	0.8232
Si1	0.5632	0.9209	0.7017
Si2	0.1352	0.9141	0.7039
C1	0.1913	0.3088	0.5301
C2	0.2956	0.6973	0.4780
O1	0.4017	0.6240	0.4441
O2	0.2796	0.6805	0.5588
O3	0.0980	0.7642	0.7269
O4	0.6595	0.0306	0.7749
O5	0.0900	0.3767	0.5699
O6	0.0993	0.0210	0.7780
O7	0.2167	0.7953	0.4285
O8	0.6376	0.7744	0.7252
O9	0.0493	0.9720	0.6027
O10	0.2745	0.2081	0.5740
O11	0.3603	0.9174	0.7254
O12	0.2028	0.3345	0.4498
O13	0.5434	0.9761	0.5994

Figure 1.- Angle-dispersive powder x-ray diffraction pattern of our sample at ambient conditions confirming the tilleyite structure (black line). A small amount of calcite was also detected. The calculated LeBail fit to the XRD integrated pattern and the profile difference are represented as red and green lines, respectively. Vertical blue and magenta lines indicate Bragg reflections of tilleyite and calcite.

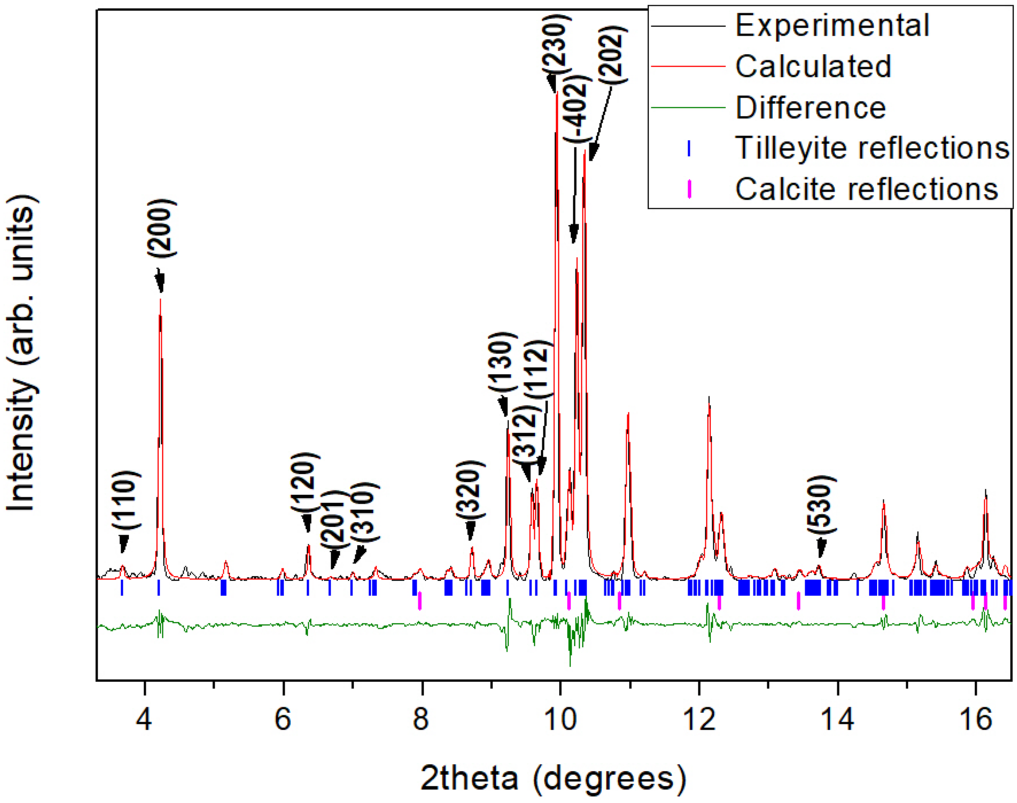

Table 3a.- Experimental details of the $Ca_5(Si_2O_7)(CO_3)_2$ tilleyite single-crystal structure refinements at 7.9 GPa

**Crystal data**	
Chemical formula	$Ca_5(Si_2O_7)(CO_3)_2$
Crystal system	Monoclinic
Space group	$P2_1/n$
Unit cell parameters	*a* = 7.4003(2) Å
	b = 9.9189(2) Å
	c = 14.573(3) Å
	β = 104.193(8)°
Cell volume	1037.0(3) $Å^3$
Z	4
Density	3.13 g/cm^3
**Crystal structure refinement**	
Total number of reflections	6667
Unique reflections	3066
Nº. refl. I > 3σ(I)	2812
R_{int}	0.0234
R1	0.0679
$R1_{all}$	0.0729
wR2	0.1625
Nº. parameters	90
GooF	1.035

Table 3b.- Atomic coordinates of tilleyite at 7.9 GPa.

Atom	x	y	z
Ca1	0.92730(10)	0.08861(6)	0.88120(11)
Ca2	0.07503(10)	0.19796(6)	0.68583(11)
Ca3	0.24690(11)	0.00898(4)	0.49658(12)
Ca4	0.06709(11)	0.57274(7)	0.63067(12)
Ca5	0.91385(11)	0.71428(7)	0.82282(12)
Si1	0.56626(14)	0.91911(9)	0.70248(16)
Si2	0.13533(9)	0.91420(6)	0.70473(16)
C1	0.1915(5)	0.2987(3)	0.5263(6)
C2	0.2916(5)	0.7051(3)	0.4827(5)
O1	0.4007(5)	0.6240(3)	0.4508(5)
O2	0.2771(5)	0.6996(3)	0.5660(6)
O3	0.1023(4)	0.7513(3)	0.7249(4)
O4	0.6677(4)	0.0267(3)	0.7801(5)
O5	0.0793(5)	0.3757(4)	0.5573(6)
O6	0.0935(5)	0.0076(3)	0.7847(5)
O7	0.2095(4)	0.7984(3)	0.4253(5)
O8	0.6372(4)	0.7679(3)	0.7233(4)
O9	0.0485(4)	0.9715(3)	0.6025(5)
O10	0.2775(4)	0.2079(3)	0.5823(5)
O11	0.3624(4)	0.9169(3)	0.7293(5)
O12	0.2074(8)	0.3013(5)	0.4414(8)
O13	0.5440(5)	0.9794(3)	0.5956(5)

Table 4.- Lattice parameters of the low-pressure phase (powder and single crystal experiments).

Pressure (GPa)	Exp.	a (Å)	b (Å)	c (Å)	β (degrees)	Volume (Å^3)
0.0001	SC-Xcalibur	7.582(4)	10.265(4)	15.030(6)	103.99(2)	1135.0(9)
0.0001	PWD-ALBA	7.575(2)	10.254(3)	14.979(4)	104.08(2)	1128.5(4)
0.0001	PWD-Rigaku	7.567(3)	10.232(3)	14.990(5)	103.94(2)	1126.5(6)
0.3	SC-PETRA	7.5663(1)	10.2293(1)	14.9958(15)	104.009(5)	1126.1(2)
0.35	PWD-ALBA	7.567(4)	10.226(5)	14.979(8)	104.07(3)	1124(1)
0.6	PWD-ALBA	7.560(4)	10.215(4)	14.977(9)	104.07(4)	1122(2)
0.8	PWD-ALBA	7.554(4)	10.215(5)	14.957(7)	104.08(4)	1119(2)
1.25	PWD-ALBA	7.549(4)	10.205(4)	14.944(9)	104.11(4)	1116(2)
1.5	PWD-ALBA	7.543(4)	10.194(4)	14.928(9)	104.12(4)	1113(2)
1.9	PWD-ALBA	7.529(4)	10.174(4)	14.887(9)	104.11(4)	1106(2)
2.5	PWD-ALBA	7.514(4)	10.152(4)	14.858(9)	104.10(4)	1099(2)
2.7	SC-PETRA	7.5098(2)	10.1281(1)	14.8412(17)	104.158(6)	1094.5(2)
3.1	PWD-ALBA	7.502(4)	10.130(4)	14.837(9)	104.16(4)	1093(2)
4.2	PWD-ALBA	7.480(4)	10.087(4)	14.763(9)	104.11(4)	1080(2)
4.8	SC-ALBA	7.4685(4)	10.0486(17)	14.702(2)	104.236(11)	1070.7(4)
5.1	PWD-ALBA	7.460(4)	10.050(4)	14.740(9)	104.15(4)	1072(2)
5.8	SC-PETRA	7.4440(1)	10.0006(1)	14.6658(15)	104.240(5)	1058.3(2)
6.3	PWD-ALBA	7.437(4)	10.011(4)	14.674(9)	104.13(4)	1059(2)
7.4	PWD-ALBA	7.410(4)	9.972(4)	14.643(9)	104.16(4)	1049(2)
7.9	SC-PETRA	7.4003(2)	9.9189(2)	14.573(3)	104.193(8)	1037.0(3)
8.2	SC-ALBA	7.4007(5)	9.918(3)	14.564(3)	104.21(2)	1036.0(7)
8.7	PWD-ALBA	7.387(4)	9.921(4)	14.557(9)	104.09(4)	1035(2)

Table 5.- Lattice parameters of the high-pressure phase (powder and single crystal experiments).

Pressure (GPa)		a (Å)	b (Å)	c (Å)	β (degrees)	Volume ($Å^3$)
9.9	PWD-ALBA	7.352(6)	9.870(4)	14.351(4)	103.93(4)	1011(2)
10.8	SC-PETRA	7.3402(11)	9.7652(7)	14.354(9)	103.88(4)	998.8(10)
12	SC-ALBA	7.3097(9)	9.790(3)	14.24(2)	104.01(2)	988(2)
11.8	PWD-ALBA	7.311(6)	9.794(4)	14.256(5)	103.88(4)	991(2)
13.5	SC-ALBA	7.2823(11)	9.750(3)	14.18(2)	104.02(2)	977(2)
13.8	PWD-ALBA	7.277(6)	9.744(4)	14.185(5)	103.89(5)	977(3)
15.1	SC-ALBA	7.257(3)	9.674(5)	14.15(2)	104.13(4)	963(2)
16.7	PWD-ALBA	7.221(7)	9.668(4)	14.097(5)	103.88(5)	955(3)
18	SC-ALBA	7.232(4)	9.617(6)	14.05(2)	104.15(4)	947(2)
18.7	PWD-ALBA	7.196(7)	9.634(4)	14.040(5)	103.94(5)	945(3)
21	SC-ALBA	7.215(4)	9.503(6)	13.98(2)	104.08(5)	930(2)
21.1	PWD-ALBA	7.168(7)	9.579(4)	13.970(5)	104.03(5)	931(3)

Figure 2.- Raman spectra of $Ca_5(Si_2O_7)(CO_3)_2$ at high pressures and room temperature.

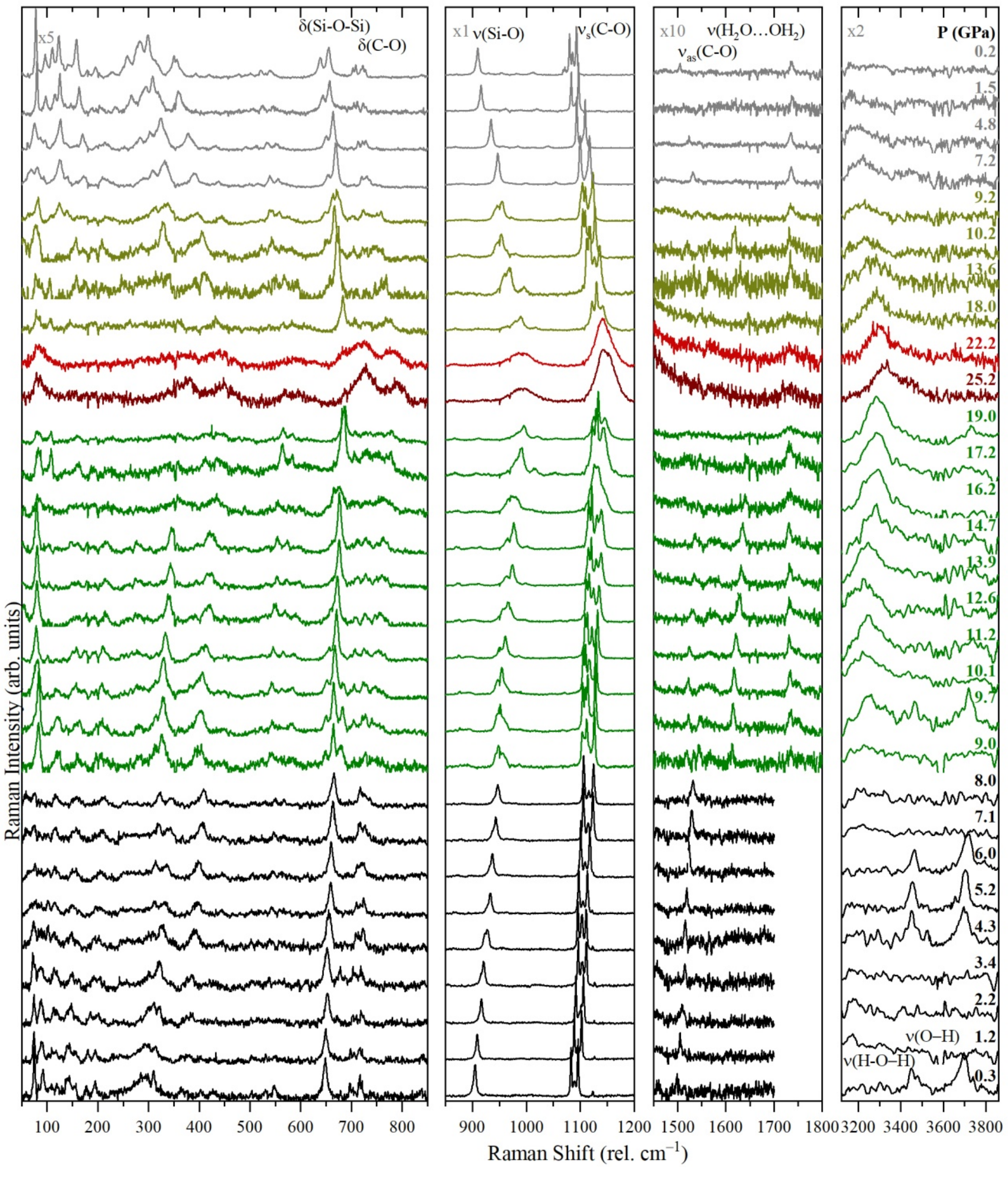

Figure 3a.- Calculated evolution of the low-frequency Raman modes of tilleyite

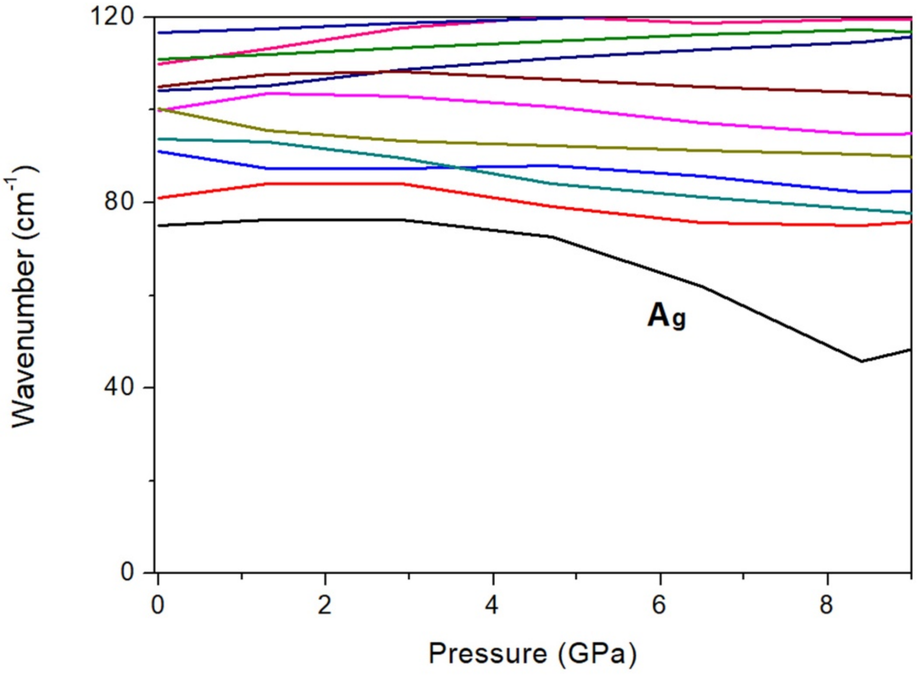

Figure 3b.- Calculated evolution of the low-frequency IR modes of tilleyite.

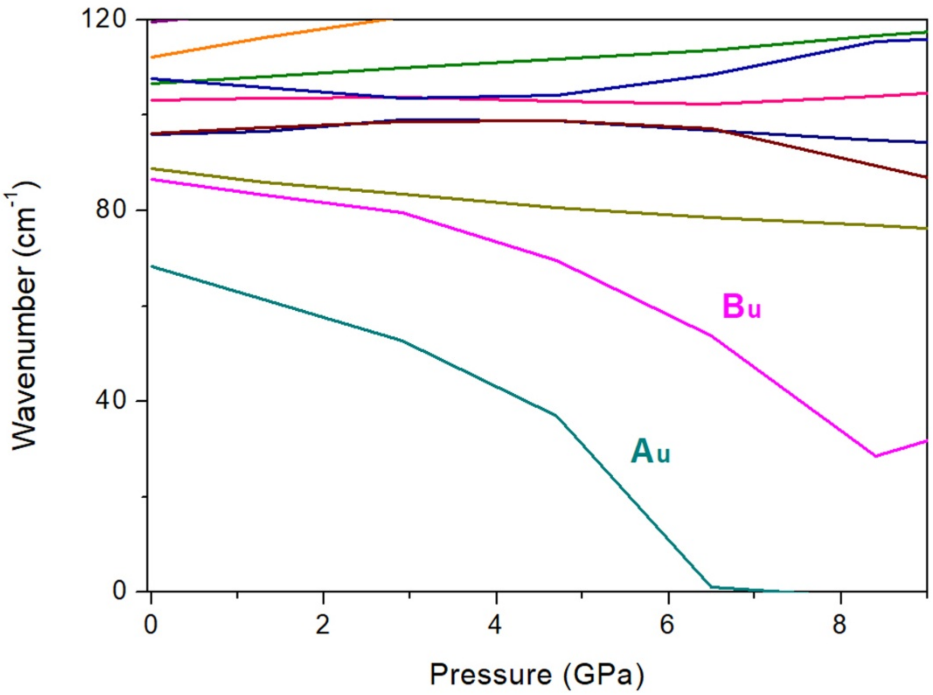

Figure 4.- Calculated evolution of the Raman and IR modes of post-tilleyite

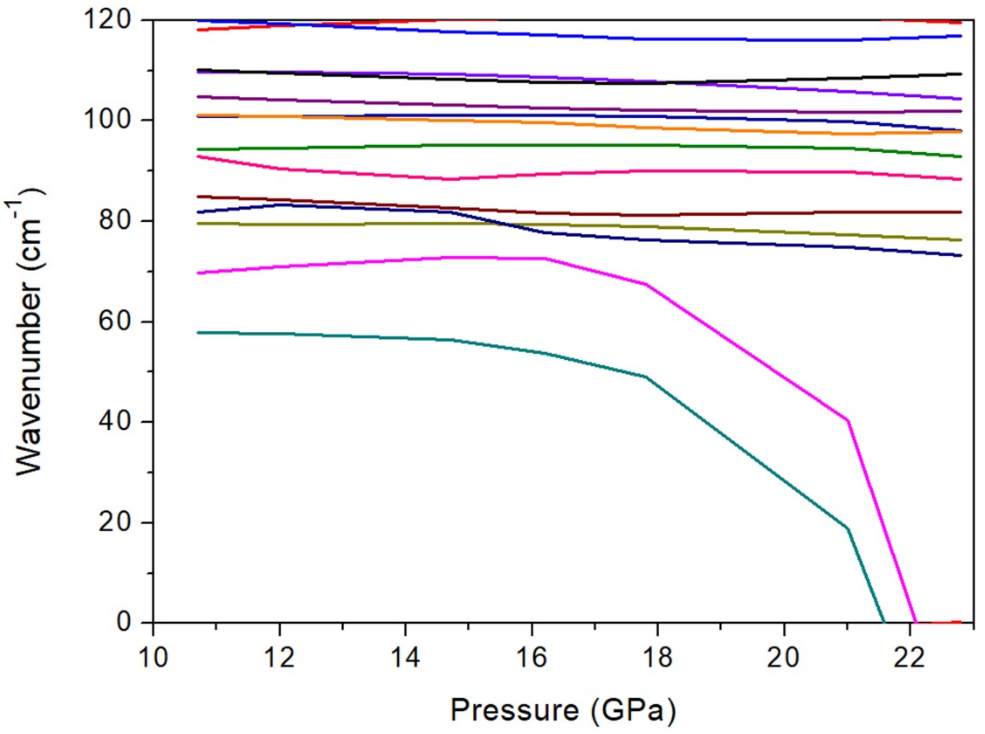

Table 6.- Bulk moduli (B_{0P}) of the different Ca-centered polyhedra in both, tilleyite and post-tilleyite structures.

Tilleyite, $P2_1/n$			Post-tilleyite, $P2_1$	
**Polyhedra**	**B_{0P} (GPa)**		**Polyhedra**	**B_{0P} (GPa)**
8				
[CaO_6]	66.2(6)		[CaO_6]	89.0(8)
[CaO_7]	58(2)		[CaO_7]	87(3)
[CaO_7]	92(2)		[CaO_7]	96(3)
[CaO_7]	101(3)		[CaO_7]	100.5(9)
[CaO_8]	85.8(7)		[CaO_8]	62.7(3)
			[CaO_8]	91.7(2)
			[CaO_8]	105.4(13)
			[CaO_8]	118(3)
			[CaO_8]	139(3)
			[CaO_9]	83.3(10)

Table 7.- High-pressure high-temperature lattice parameters of the low-pressure tilleyite phase (from powder XRD data measured at ALBA).

Pressure (GPa)	Temperature (ºC)	a (Å)	b (Å)	c (Å)	β (degrees)	Volume ($Å^3$)
1E-4	22	7.584(3)	10.247(4)	14.996(8)	104.10(3)	1130(1)
0.3	45	7.584(3)	10.240(4)	14.979(8)	104.11(4)	1128(2)
0.9	58	7.574(4)	10.221(5)	14.961(8)	104.07(4)	1123(2)
1.7	100	7.564(4)	10.200(5)	14.923(8)	104.05(4)	1117(2)
2.4	134	7.555(4)	10.179(5)	14.879(8)	104.06(4)	1110(2)
3.3	179	7.537(4)	10.133(5)	14.846(8)	104.15(4)	1100(2)
4.4	260	7.518(4)	10.099(5)	14.804(8)	104.10(4)	1090(2)
5.0	300	7.492(4)	10.075(5)	14.786(9)	104.05(4)	1083(2)
5.4	336	7.496(5)	10.065(5)	14.765(8)	104.07(4)	1080(2)
6.0	376	7.485(4)	10.054(5)	14.747(8)	104.09(4)	1076(2)
6.6	401	7.478(5)	10.036(5)	14.726(8)	104.23(4)	1071(2)
7.2	402	7.458(5)	10.019(5)	14.689(9)	104.21(4)	1064(3)

Figure 5.- Solid symbols indicate the difference between measured high-temperature volume for $Ca_5(Si_2O_7)(CO_3)_2$ tilleyite and room-temperature reference volume (RT-EOS), divided by the measured volume at each pressure, as a function of temperature. The slope of the linear fit (red line) provides an estimation of the average thermal expansion.

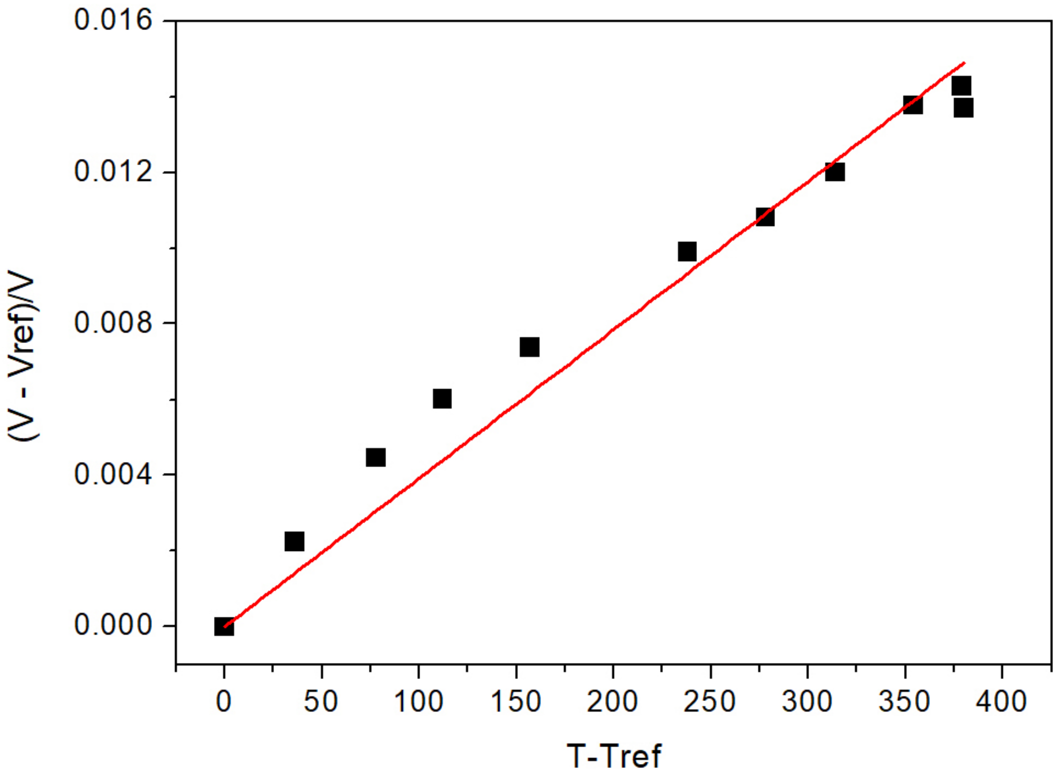

Supplement: Supplementary file 1 — Supplementary information [file 41598_2019_44326_MOESM1_ESM.pdf]
